# Asymptomatic Hypotension in a Patient with Catheter-related Right Atrial Thrombus

**DOI:** 10.5811/cpcem.2017.10.35608

**Published:** 2018-01-09

**Authors:** Hillary E. Davis, Michael Lu, Scott J. Cameron, Ryan Bodkin

**Affiliations:** * University of Rochester Medical Center, Department of Emergency Medicine, Rochester, New York; † University of Rochester Medical Center, Department of Cardiology, Rochester, New York

## Abstract

Atrial thrombi can be a complication in patients with indwelling central-line catheters, and failure to diagnose can potentially be lethal. This condition is generally associated with profound hypo-perfused states. Here we present a case of a 77-year-old female who arrived to our emergency department for evaluation of a leg laceration and was incidentally found to have a catheter-related right atrial thrombus using point-of-care ultrasound.

## INTRODUCTION

Long-term central venous catheters are often necessary for patients with chronic illnesses, providing repeated access for hemodialysis, blood tests, parenteral nutrition or delivery of intravenous medications. Thrombosis is a relatively common complication of central-venous indwelling catheters secondary to the turbulence their geometry invokes on the typical laminar adjacent flow.^1^ Without intervention, thrombi can lead to vascular compromise by either growing substantial enough to occlude its native vessel or embolizing to another. Treatment either by anticoagulation therapy or systemic thrombolysis, or surgical thrombectomy is necessary to prevent acute decline. Patients typically present to the emergency department (ED) with overt signs of end-organ hypoperfusion. Here we present a rare case of an incidentally found catheter-related right atrial thrombus (CRAT).

## CASE REPORT

A 77-year-old female presented to our ED from her assisted living center for evaluation of a right lower leg laceration. One hour prior to arrival, she struck her leg against her walker when attempting to use the toilet and sustained a laceration to the skin overlying her anterior tibia. She was brought immediately to the ED for evaluation. She had been in her usual state of health prior to this episode. Her comorbidities included end stage renal disease (ESRD) on dialysis, hypertension, hypothyroidism, pulmonary embolism and pulmonary hypertension. She required two liters (L) of oxygen via nasal cannula at baseline, and her warfarin dosing had not changed for over six months. She had received her full course of dialysis the day prior through her 14-month-old right subclavian catheter without any incidents.

Upon emergency physician (EP) evaluation, she endorsed minimal pain at the wound. There was no active bleeding observed. She was afebrile (37.5 degrees Celsius) with a heart rate of 90 beats/minute, respiratory rate of 18 breaths/minute, blood pressure of 77/59 millimeters of mercury (mmHg), and an oxygen saturation of 93% on her home two L of oxygen. She denied any shortness of breath, palpitations, lightheadedness or chest pain. She continued to deny such even during periods of exertion such as ambulating, and she required no further increase in her oxygen level. Per prior documentation, her baseline systolic blood pressure ranged between 90 to 100 mmHg. Subsequent blood pressures over the following two hours included systolic blood pressures in the 50s mmHg; she continued to remain asymptomatic.

**Image 1:**
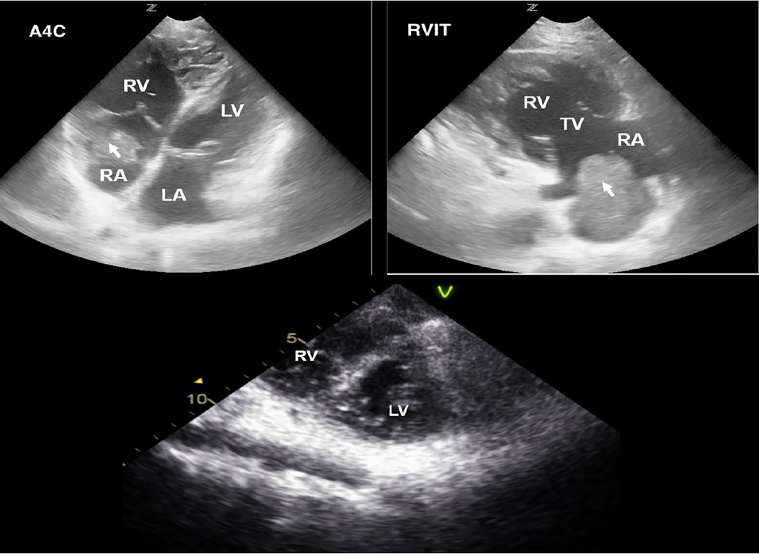
Top: Point-of-care ultrasound echocardiogram. (left: apical 4-chamber view; right: right ventricle inflow tract view); right atrial mass is noted by the arrowhead. Bottom: Formal transthoracic echocardiogram, parasternal short- axis view, enlarged right ventricle with intraventricular septum flattening in diastole consistent with pressure overload. *LA*, left atrium; *RA*, right atrium; *LV*, left ventricle; *RV*, right ventricle; *TV*, tricuspid valve.

The patient’s wound was closed without incident. She was given a 500 milliliter bolus of normal saline and five milligrams of midodrine without any noticeable effect on her blood pressure. Subsequently ordered diagnostic studies were notable for an international normalized ratio (INR) of 2.9, a hematocrit of 31%, white blood count 14.8×10^9^/L, lactate 1.7 millimoles/L and an anion gap of 13 milliequivalents/L. Electrolytes were grossly normal. The EP performed a point-of-care ultrasound (POCUS) echocardiogram to evaluate for a uremic pericardial effusion. A mass was observed in the right atrium on multiple views (Image 1).

**Image 2:**
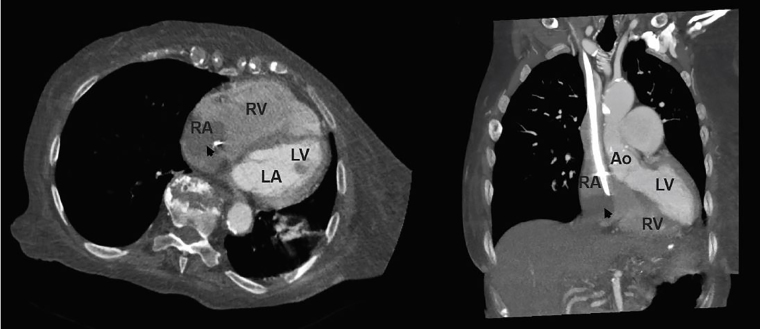
Computed tomography angiogram of the chest. Left (cross section) Right (coronal section). Right atrial mass is noted by the arrowhead. Dialysis catheter tip noted as a bright white artifact within the thrombus. *LA*, left atrium; *RA*, right atrium; *LV*, left ventricle; *RV*, right ventricle; *Ao*, aorta.

Subsequently, a computed tomography angiogram chest demonstrated a catheter-associated right atrial thrombus along with a right lower lung pulmonary embolism (Image 2). Cardiology was called to evaluate the patient and suggested that her hypotension might be secondary to the reduced inflow into the right ventricle resulting in left ventricle underfilling. A complete echocardiogram demonstrated a reduced left ventricular end diastolic volume with normal ejection fraction between 55–75%, a stroke volume of 29.36 liters per square meters and a cardiac output of 4.27 liters per minute (L/M) (normal 5–7 L/M). The right ventricle ejection fraction was moderately reduced with right ventricular enlargement and septal flattening: “a positive D-sign.” A tissue density mass measuring 4.0 × 3.7 centimeters attached to the right atrial free wall was also seen. An agitated saline study was negative for a right-to-left intracardiac shunt. Compared to a prior echocardiogram performed eight months earlier, there was an interval increase in estimated pulmonary artery pressures (from 60 to greater than 92 mmHg) and a new right atrial mass. Upon further investigation, the EP learned that the patient had recently changed her diet and she’d had one subtherapeutic INR one month prior.

The atrial thrombus was deemed too large for percutaneous suction thrombectomy. Patient declined surgical thrombectomy after a cardiac surgeon explained that her risk of mortality was extremely elevated. She was medically managed in the cardiac intensive care unit with a heparin drip and phenylephrine. Her pressor support was weaned and her blood pressure was 119/49 mmHg at the time of discharge. She returned to her assisted living center on midodrine and without any changes in her warfarin on hospital day 14.

## DISCUSSION

We present a case of CRAT that was discovered incidentally by an EP while investigating the patient’s asymptomatic hypotension. By detecting this pathology, the patient’s course of care was significantly altered from what she might otherwise have experienced if she had been solely evaluated for her chief complaint.

CPC-EM Capsule

What do we already know about this clinical entity?

Patients with central venous catheters are at risk for catheter-related right atrial thrombus (CRAT) and their associated complications such as pulmonary embolism, arrhythmias, and septic emboli.

What makes this presentation of disease reportable?

This case of CRAT was discovered secondary to the patient’s asymptomatic hypotension leading to the finding of the patient being in cardiogenic shock.

What is the major learning point?

Always be suspicious of CRAT in patients with central venous catheters and do not ignore vital sign abnormalities.

How might this improve emergency medicine practice?

Emergency physicians might consider using point-of-care ultrasound echocardiograms more frequently for potential visualization of right atrial thrombi in patients with central venous catheters.

The incidence of CRATs is not inconsequential. One prospective autopsy study demonstrated that mural thrombi were present in 29% of central lines.^2^ Catheters placed in the right atrium are particularly sensitive to thrombus formation and patients may not present with symptoms.^3^ This is particularly concerning as mortality from CRAT is high, ranging from 18% to 47%, and can be secondary to pulmonary embolism, arrhythmias, septic emboli, or even therapeutic interventions.^4^,^5^ CRATs are considered type B thrombi, meaning that they originate in the atrium itself and are usually secured to the atrial wall. This contrasts with their type A counterparts, which originate in the deep peripheral veins and mobilize, typically resulting in systemic emboli. Albeit pulmonary emboli are less likely to occur with type B thrombi, the simultaneous presence of a pulmonary embolism with an atrial thrombus is often a worse prognostic sign.^6^ Fourteen percent of hemodialysis-related CRATs will also have concomitant pulmonary emboli.^7^ However, a single pulmonary embolism in the right lower lobe, as in this case, would not likely be responsible for significantly decreasing left ventricle preload leading to the patient’s profound hypotension.

Our patient experienced altered preload from right atrial obstruction consequent to the CRAT’s substantial size. She was likely asymptomatic secondary to the slow accumulation of thrombus burden, providing time for the right heart to adapt. Further, her history of pulmonary hypertension would lead to a baseline of right heart strain and would likely mask new symptoms resulting from CRAT growth. A similar case of “compensated shock” secondary to type A right heart thrombi has been reported previously in which a 50-year-old male was evaluated for bilateral leg pain and, due to his asymptomatic tachycardia, a POCUS echocardiogram was performed leading to the ultimate diagnosis.^8^

**Image 3:**
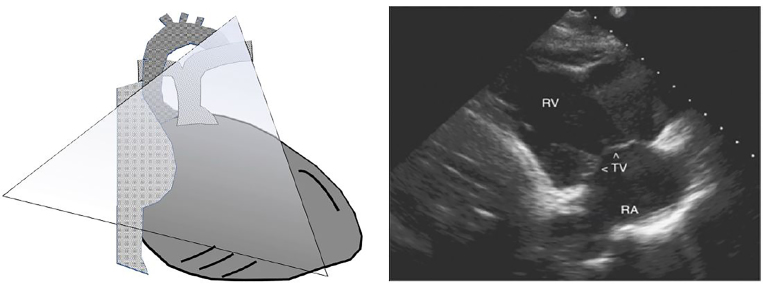
Right ventricle inflow tract view. Left: Graphical representation. Right: Point-of-care ultrasound with normal anatomy. *RA*, right atrium; *RV*, right ventricle; *TV*, tricuspid valve.

The right atrium is not typically the focus of POCUS echocardiography, and is usually only partially visualized on the subxyphoid, apical-4-chamber (A4C), or high parasternal short views. Our patient’s right atrial thrombus was identified on the right ventricle inflow tract (RVIT) view, which provides a more dedicated assessment of the right heart chambers and tricuspid valve. This view is obtained by placing the ultrasound probe in the parasternal long-axis position and tilting the beam anteriorly toward the chest wall (Image 3). Note the dilated, enlarged appearance of our patient’s right ventricle in comparison to a normal RVIT appearance. Another important thing to point out is the appearance of the ventricles in the A4C view, as a dilated hypertrophied right ventricle may be mistaken for the left ventricle, especially if the probe is flipped. In addition to double-checking the machine settings and probe marker position, identifying the left ventricular outflow tract on the image and paying attention to which side it originates can help the POCUS provider differentiate between the right and left cardiac chambers.

Although the patient in this case was asymptomatic, it can be rationalized that she was in cardiogenic shock. By strict definition, cardiogenic shock is defined as a low systolic blood pressure (<90 mmHg) in combination with a reduced cardiac output and adequate or elevated pulmonary wedge filling pressures.^9^ Per the patient’s complete echocardiogram, her cardiac index would not meet the criteria to be considered for cardiogenic shock. However, a right heart catheter using the thermodilution method is the most accurate method to discern cardiac index, as two-dimensional echocardiograms have been found to be a less reliable substitute.^10^

The Shock Index (SI = heart rate/systolic blood pressure) has been employed successfully as a means to predict mortality in patients with cardiogenic shock and in a general ED patient population.^11^–^13^ Values for a SI ranging from 0.5–0.7 beats/minute/mmHg are considered normal, while values above 1.2 beats/minute/mmHg are associated with increased mortality. Our patient presented with a SI of 1.2 beats/minute/mmHg, which increased to 1.8 beats/minute/mmHg during her ED evaluation. Perhaps in the absence of pulmonary capillary wedge pressures, the SI can be employed in the ED to determine patients at risk for cardiogenic shock; however, further prospective trials are necessary for validation.

## CONCLUSION

This is not the first case reported in literature using POCUS echocardiograms for visualization of right atrial thrombi; when used previously, the patients were in obvious states of end-organ hypoperfusion.^8^,^14^ Here we present an unusual presentation of CRAT in an asymptomatic patient that highlights the importance of noting abnormalities in vital signs. Given CRAT’s high potential for morbidity and the relatively low risk and ease of obtaining a POCUS echocardiogram, we advocate that patients with a right heart catheter and vital sign abnormalities not attributed to another pathological state be evaluated in the ED for CRAT with a screening POCUS.
